# Statistical Visibility of Curated TF–Target Regulatory Relationships and Reverse Consistency of Top-Ranked TF–Gene Pairs in Single-Cell Expression Data

**DOI:** 10.3390/genes17091110

**Published:** 2026-09-12

**Authors:** Wenqing Feng, Zejun Zhang, Zheng Wu, Chengyu Yuan, Jinlei Sun, Guoqiang Wang, Yunqing Liu

**Affiliations:** 1 School of Computer Science, Luoyang Institute of Science and Technology, Luoyang 471000, China; 18737700353@163.com (W.F.); 17805925415@163.com (Z.W.); yuancy@lit.edu.cn (C.Y.); jlsun@lit.edu.cn (J.S.); wgq@lit.edu.cn (G.W.); 2 School of Electrical and Energy Engineering, Shanghai Dianji University, Shanghai 201306, China; 18734968335@163.com

**Keywords:** single-cell RNA sequencing, TF–target regulatory relationships, statistical visibility, matched background pairs, reverse consistency, pathway coherence

## Abstract

Background/Objectives: Large language models and automated analytical tools show potential for biomedical text understanding, knowledge integration, and single-cell data interpretation, but interpretations of specific gene regulatory relationships still require empirical grounding. For TF–target relationships, one relevant constraint is whether curated regulatory edges show detectable expression-level statistical evidence in the single-cell data being interpreted, because such evidence may vary across cellular states, conditions, and regulatory mechanisms. We therefore evaluated the statistical visibility of known transcription factor (TF)–target relationships in single-cell expression space to aid the interpretation of regulatory inference and automated-tool outputs. Methods: We performed two complementary analyses: first, testing whether curated TF–target edges showed stronger pair-level associations than matched background gene pairs; and second, assessing whether high-scoring TF–gene pairs corresponded to existing regulatory knowledge and whether their target genes showed pathway coherence. Results: Across four peripheral blood mononuclear cell (PBMC) datasets, four adult tissues, and seven adult cell types, curated TF–target relationships showed weak but reproducible statistical visibility rather than strong separation. For all five association metrics, the mean area under the precision–recall curve (AUPRC) was only slightly above the random-ranking baseline of 0.5. High-scoring pairs were more often supported by curated resources, and their target genes showed context-specific Hallmark pathway coherence. Conclusions: Single-cell expression associations can provide useful but limited statistical clues for TF–target regulation and should be interpreted as complementary rather than definitive regulatory evidence.

## 1. Introduction

Regulatory relationships between transcription factors (TFs) and target genes are central to understanding the maintenance of cellular identity, responses to stimulation, and the execution of biological programs [1]. TFs can influence downstream gene expression through direct or indirect mechanisms, and many TF–target relationships have been compiled in regulatory databases through experimental studies, literature curation, and integrative analyses. These curated regulatory resources provide an important reference for examining how transcriptional regulatory relationships are reflected in expression data.

Single-cell RNA sequencing (scRNA-seq) enables gene expression patterns to be examined at cellular resolution and captures heterogeneity across cell types, cell states, and stimulation conditions [2]. This creates an opportunity to assess whether TF–target regulatory relationships supported by prior biological evidence can be detected in single-cell expression space. Reproducible expression-level statistical signals from known TF–target relationships may help interpret single-cell expression associations and prioritize regulatory hypotheses for downstream analysis.

However, regulatory relationships are not necessarily visible in expression matrices. TF mRNA abundance does not always reflect protein abundance [3], and post-translational modifications may further alter TF activity [4]. Chromatin accessibility, cofactor availability, temporal delays between regulatory activity and mRNA expression, and the sparsity and condition dependence of single-cell expression matrices [5] may also weaken expression-level associations. Thus, even biologically supported TF–target relationships may show only weak expression associations in a given single-cell dataset. Moreover, some highly correlated TF–gene pairs may reflect co-expression, shared pathway activation, or differences in cellular composition rather than direct regulation.

As large language models and domain-tool-augmented systems are increasingly used for biomedical knowledge access and hypothesis generation, automated interpretations of gene regulatory relationships require grounding in reliable domain evidence [6,7]. For TF–target relationship interpretation, text-level knowledge retrieval, curated database annotations, and empirical evidence from single-cell expression data represent related but distinct sources of support. Curated regulatory resources provide structured prior knowledge, whereas single-cell expression matrices determine whether these prior relationships leave observable expression-level statistical traces in specific biological contexts. Accordingly, this study treated curated regulatory databases as external biological knowledge and evaluated whether their TF–target relationships retained reproducible expression-level statistical traces under matched evaluation conditions.

This study evaluated the statistical visibility of curated TF–target relationships in single-cell expression data. We used regulatory edges from established curated databases and analyzed multiple single-cell datasets spanning peripheral blood mononuclear cell (PBMC), adult tissue, and adult cell-type contexts. Through two complementary analytical directions, the study provides an empirical reference for interpreting TF–target expression associations in single-cell transcriptomes. The analysis focused on whether curated regulatory relationships retained measurable, reproducible statistical signals under real single-cell data conditions. This perspective helps clarify the value of single-cell expression data as a source of regulatory clues while defining the interpretive boundaries of mRNA-based association analysis.

## 2. Materials and Methods

### 2.1. Study Design

**Figure 1:**
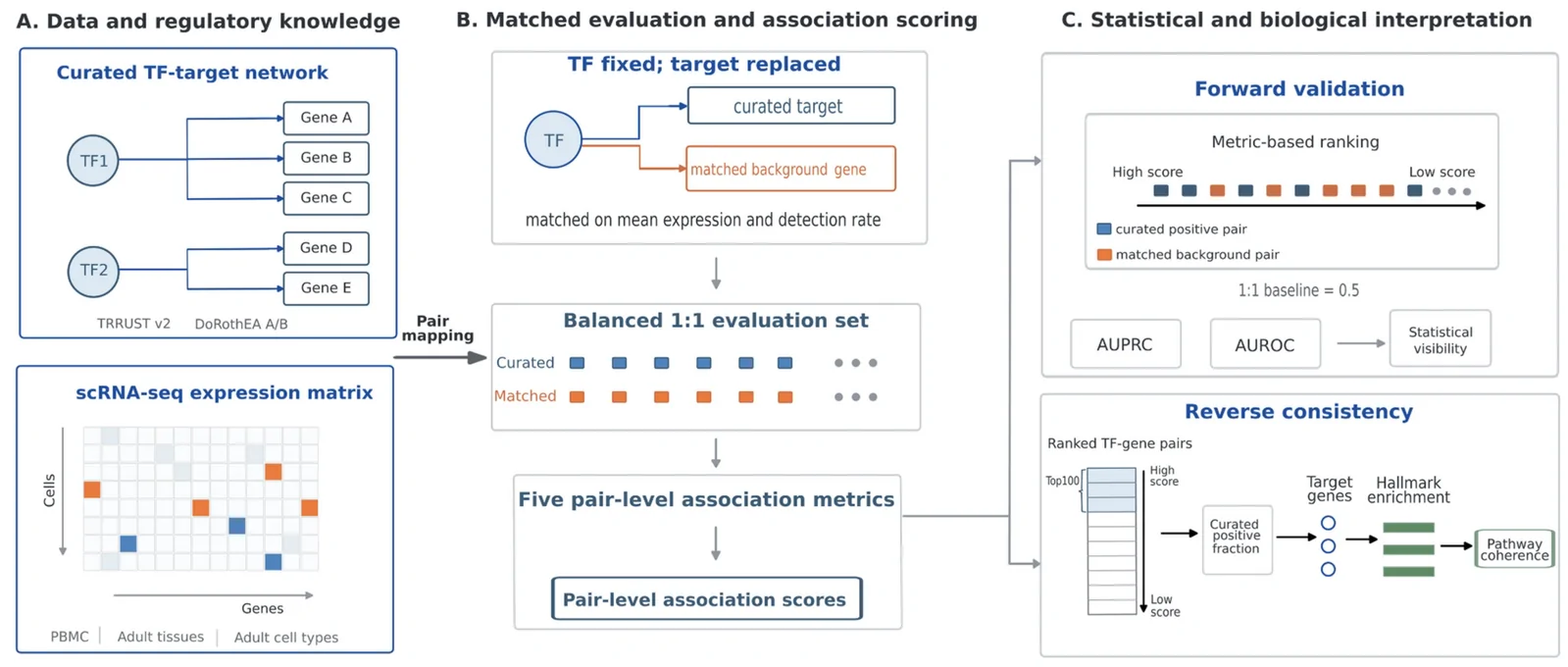
Overview of the framework used to evaluate the statistical visibility of curated TF–target relationships in single-cell expression data. Curated relationships from TRRUST v2 and DoRothEA A/B were mapped to expression matrices from PBMC, adult tissue, and adult cell-type contexts. For each curated pair, the TF was retained and a target gene with similar mean expression and detection rate was selected to form a matched background pair, thereby constructing a balanced 1:1 evaluation set. Five pair-level association metrics were used for forward validation and reverse consistency analysis. Forward validation used AUPRC and AUROC to assess the ranking of curated pairs relative to matched pairs. Reverse consistency quantified the curated-positive fraction among fixed Top100 pairs, defined as the 100 highest-scoring TF–gene pairs within each matched evaluation set for a given metric, and summarized Hallmark enrichment among their target genes. Under the balanced design, the random AUPRC baseline and the baseline curated-positive fraction were both 0.5. The random AUROC baseline was also 0.5 but did not depend on class prevalence. In the schematic scRNA-seq expression matrix, colored squares illustrate gene expression signals. The colors are schematic and do not represent quantitative expression levels or distinguish curated-positive from matched-background pairs.

The study workflow comprised two related analyses. In forward validation, curated TF–target edges were combined with scRNA-seq expression matrices to construct balanced 1:1 evaluation sets containing curated-positive pairs and matched background pairs. Background pairs were generated by retaining the TF and selecting an alternative target gene with similar mean expression and detection rate. Five pair-level association metrics were then calculated. The area under the precision–recall curve (AUPRC) and the area under the receiver operating characteristic curve (AUROC) were used to assess whether curated edges tended to rank above matched background pairs. In reverse consistency analysis, the same precomputed scores were used to rank the evaluated TF–gene pairs, quantify the curated-positive fraction among high-scoring pairs, and examine pathway coherence among the target genes of high-scoring curated-positive pairs. Analyses were performed separately within each condition and regulatory edge set. These condition × edge-set combinations formed the lowest computational evaluation level. Their estimates were subsequently summarized within the corresponding dataset, tissue, or cell type and then used for cross-context comparisons. Edge sets and matching repeats were not treated as biological replicates. The overall study workflow is summarized in Figure 1.

### 2.2. Data Sources and Curated Regulatory Edges

Curated TF–target relationships were obtained from TRRUST v2 [8] and the A/B confidence levels of DoRothEA [9]. TRRUST v2 provides manually curated transcriptional regulatory interactions derived from published literature, whereas DoRothEA integrates multiple evidence sources, including literature-curated resources, ChIP-seq evidence, motif-based information, and gene-expression-based inference, and assigns confidence levels to TF–target interactions. The A/B confidence levels of DoRothEA were used to prioritize higher-confidence regulatory edges. These two resources were selected because they provide structured and evidence-supported prior TF–target annotations that are independent of the single-cell expression matrices analyzed in this study. Gene symbols were standardized before analysis, and directed TF–target combinations were deduplicated. Four regulatory edge sets were defined: TRRUST, DoRothEA A/B, their intersection, and their union. The intersection was a conservative consensus subset supported by both resources, whereas the union provided broader curated coverage. The Kang IFN-β PBMC and GSE178429 IFN-γ PBMC datasets both included control and interferon-stimulated conditions. In the current analysis, these two datasets were evaluated only with the conservative consensus intersection shared by TRRUST and DoRothEA A/B; control and stimulated conditions were assessed separately using the same edge set. This design evaluated the statistical visibility of the same consensus network across conditions and across the two PBMC datasets. PBMC10k, NYGC multimodal PBMC, and the Human Cell Landscape (HCL) adult tissue and cell-type contexts were evaluated with all four predefined edge sets to assess robustness to the definition of the prior network.

### 2.3. Single-Cell Datasets and Gene Mapping

The study included four PBMC datasets: PBMC10k [10], NYGC multimodal PBMC [11], Kang IFN-β PBMC [12], and GSE178429 IFN-γ PBMC [13]. The raw GSE178429 data were obtained from the Gene Expression Omnibus [14]. Four adult tissues and seven adult cell types from the Human Cell Landscape were also included [15]. The complete dataset, conditions, and edge-set coverage are provided in Supplementary Table S1. Control and stimulated conditions in Kang and GSE178429 were first evaluated separately and then combined to form one final biological context for each dataset before inclusion in subsequent cross-context summaries. The lowest-level analysis unit was one condition × edge-set evaluation. The combinations of available conditions and edge sets across contexts yielded 56 units, which were further summarized into 15 final biological contexts. Correlation and mutual information metrics used normalized log expression, whereas detection-based metrics and matching variables used detection states derived from raw counts. Gene symbols were standardized, and a regulatory edge was retained only when both the TF and target mapped to the expression matrix and were detected in at least 5% of cells.

### 2.4. Construction of Strictly Matched Background Gene Pairs

Forward validation used a strict matched-background design to control for biases related to expression level and detection rate. For each curated TF–target edge, the TF was retained and only the target gene was replaced. Within each condition × edge-set evaluation, a candidate background target was required to differ from the curated target by no more than 0.25 standard deviations (SD) for both standardized mean raw count and standardized detection rate. In each matching repeat, eligible candidates were ranked by distance, and closer candidates were preferentially selected to construct background pairs with similar expression characteristics.

Candidate background pairs involving the TF itself, the original target, a curated target of the same TF, a duplicate TF–gene combination within the same repeat, or an overlap with the union of curated edges were excluded. Pair uniqueness was enforced within each repeat, and only positive edges successfully matched in all 10 repeats were retained. A positive edge with no eligible background target in any repeat was removed from the matched evaluation set. The final analysis reported the number of retained positive edges, matching coverage, and repeat-level validity. Detailed quality-control results are provided in Supplementary Table S2.

### 2.5. Pair-Level Association Metrics

Five pair-level association metrics were used to quantify statistical dependence between TF and target-gene expression in single-cell matrices. Because the analysis focused on direction-agnostic expression-level association rather than the direction of activation or repression, Pearson and Spearman correlation coefficients were converted to absolute values. The metrics were defined as follows.

Absolute Pearson correlation measured direction-independent linear association between the expression vectors of a TF and its target gene.

(1) $$s_{Pearson}(t,g)=|{cor}_{Pearson}(x_{t},x_{g})|$$

Here, $x_{t}$ and $x_{g}$ denote the expression vectors of TF *t* and target gene *g*, respectively, across all cells.

Absolute Spearman correlation measures the direction-independent rank-based association between TF and target-gene expression.

(2) $$s_{Spearman}(t,g)=|{cor}_{Spearman}(x_{t},x_{g})|$$

Spearman correlation was calculated from ranked expression values, and its absolute value was used in the formal analysis.

Mutual information (MI) measured nonlinear dependence between the expression distributions of a TF and its target gene. In the formal analysis, the expression ranks of both genes were first discretized into eight bins before MI was calculated [16].

(3) $$MI(t,g)=\sum_{i,j}p_{ij}log\left(\frac{p_{ij}}{p_{i}p_{j}}\right)$$

Here, $p_{ij}$ denotes the joint probability that the TF and target gene fall into expression bins *i* and *j*, respectively, and $p_{i}$ and $p_{j}$ denote the corresponding marginal probabilities.

Co-expression probability measures the fraction of cells in which both the TF and target gene were detected, with detection defined as a raw count > 0.

(4) $$P_{coexpr}(t,g)=\frac{n_{11}}{N}$$

Here, *N* denotes the total number of cells, and $n_{11}$ denotes the number of cells in which both the TF and target gene were detected, with detection defined as a raw count > 0.

The co-detection odds ratio measured enrichment for joint detection of a TF and its target gene. A 2 × 2 contingency table was constructed from the detected/undetected states of the two genes.

(5) $$OR(t,g)=\frac{\left(n_{11}+0.5)(n_{00}+0.5\right)}{\left(n_{10}+0.5)(n_{01}+0.5\right)}$$

Here, $n_{11}$ denotes the number of cells in which both genes were detected, $n_{10}$ the number in which only the TF was detected, $n_{01}$ the number in which only the target gene was detected, and $n_{00}$ the number in which neither gene was detected. The 0.5 term was used as a continuity correction to prevent an undefined odds ratio when any cell of the contingency table was zero [17].

### 2.6. Statistical Evaluation of Forward Validation

Forward validation assessed whether curated-positive pairs tended to receive higher scores than strictly matched background pairs across the five pair-level metrics. The primary evaluation measures were AUPRC, AUROC, and Cliff’s delta. Precision–recall curves summarized the performance of positive pairs in the high-scoring region of the ranking [18]. Receiver operating characteristic curves evaluated discrimination across the full ranking [19]. Cliff’s delta was used as a nonparametric effect-size measure [20]. Because each repeat contained equal numbers of curated-positive and matched background pairs, the balanced random baseline for AUPRC was 0.5. An AUPRC or AUROC above 0.5 was interpreted as evidence that curated edges tended to rank above matched background pairs for that metric, not as evidence of causal regulation. For cross-context summaries, values were first summarized within each final biological context and then averaged with equal weight across contexts within each group. Condition × edge-set evaluations were computational analysis units, not biological replicates. The 10 matching repeats altered only the specific strictly matched background pairs and were used to assess whether results depended on a particular set of background pairs; they were not used to estimate biological sampling uncertainty.

For each pair-level metric, TF–gene pairs were ranked from highest to lowest score. AUPRC was calculated as average precision using Equation (6). Curated TF–target edges were labeled as positive, whereas matched background pairs were used as comparison pairs and were not regarded as validated nonregulatory edges.

(6) $$AUPRC=\sum_{n}(R_{n}-R_{n-1}){\;P}_{n}$$

Here, $P_{n}$ and $R_{n}$ denote precision and recall, respectively, at the *n*th threshold in the ranked list. Because curated-positive and matched background pairs were included at a 1:1 ratio, the expected AUPRC under random ranking was 0.5.

### 2.7. Reverse Consistency and Pathway Analysis

Reverse consistency analysis was restricted to the matched evaluation sets and did not expand to an unrestricted TF-by-gene search space. Within each condition × edge-set × repeat × metric combination, positive and background pairs were ranked by their precomputed metric scores, and the curated-positive fraction among high-scoring pairs was calculated. Because every evaluation set contained equal numbers of positive and background pairs, the balanced baseline for the curated-positive fraction was 0.5. The primary reverse-consistency analysis used a fixed Top100 threshold, defined as the 100 highest-scoring TF–gene pairs within each matched evaluation set for a given metric; evaluations with fewer than 100 ranked pairs were set to missing at this threshold and were excluded from Top100 averages. Two additional ranking thresholds were used as sensitivity analyses, with results in Supplementary Table S3: the strict Top500 threshold retained the 500 highest-scoring TF–gene pairs within a matched evaluation set and was calculated only when at least 500 ranked pairs were available, whereas the top-5% threshold retained the highest-scoring 5% of TF–gene pairs within each matched evaluation set and therefore used a context-specific top-*N*. Hallmark gene sets were obtained from the Molecular Signatures Database (MSigDB) [21]. Pathway enrichment used target genes from high-scoring curated-positive pairs, with the analyzable target genes in the corresponding evaluation as the background [22]. Enrichment was tested using a one-sided hypergeometric test [23]. Multiple testing was controlled using the Benjamini–Hochberg false discovery rate (FDR) procedure [24], applied across all tested Hallmark gene sets within each condition × edge-set × repeat × metric combination. In each biological context, Hallmark enrichment was evaluated independently for the target genes of high-scoring curated-positive pairs.

## 3. Results

### 3.1. Curated TF–Target Edges Retain Statistical Visibility Under Strict Matching

**Figure 2:**
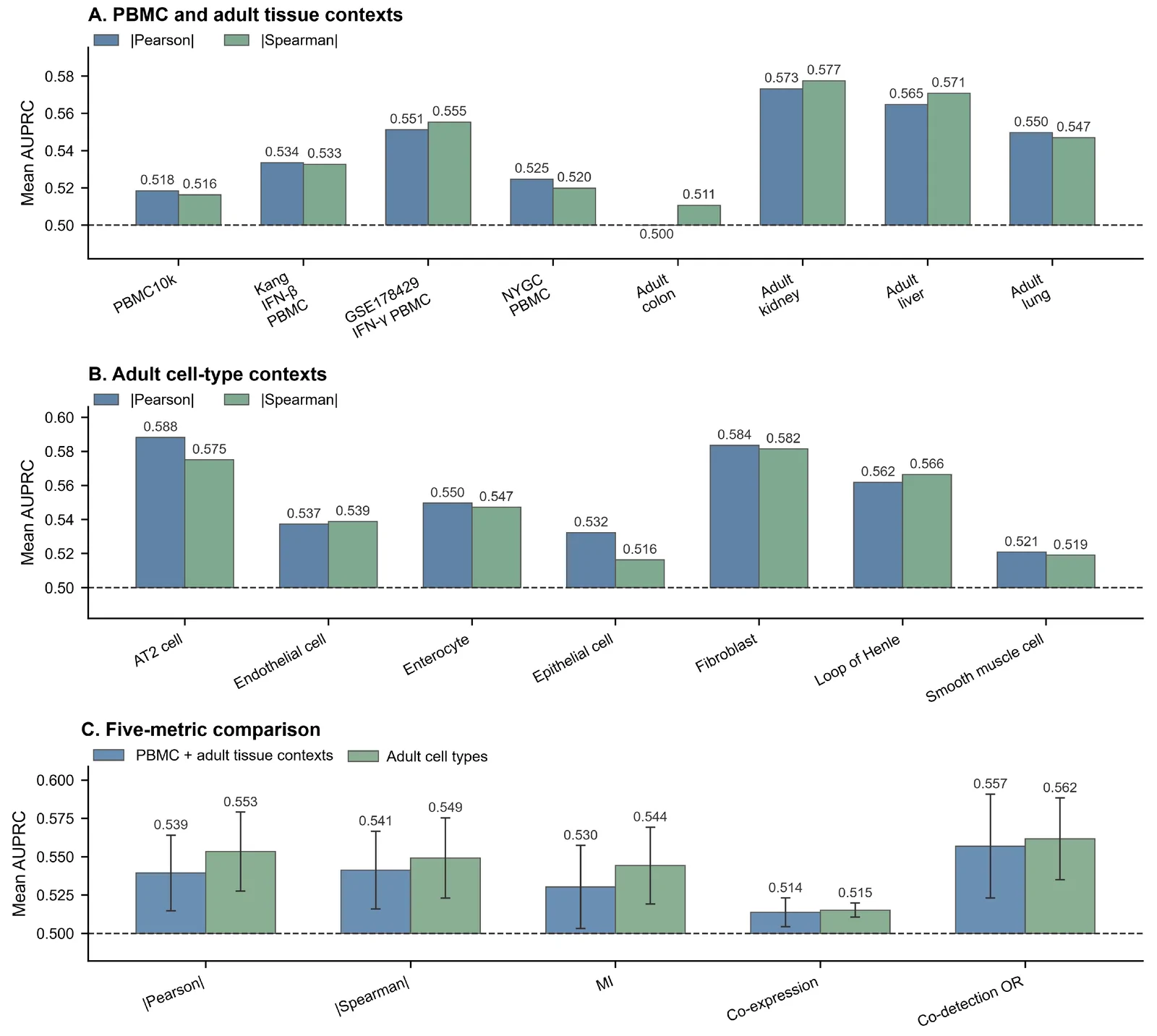
Forward visibility of curated TF–target edges across biological contexts. Panel (**A**) shows mean AUPRC values for absolute Pearson and absolute Spearman in PBMC and adult tissue contexts; Panel (**B**) shows the same two metrics in adult cell-type contexts. To maintain readability at the context level, the first two panels display only these two correlation metrics. Panel (**C**) reports the equal-weight mean AUPRC across final biological contexts for all five pair-level metrics in PBMC plus adult tissue contexts and adult cell types. The dashed line indicates the expected balanced baseline of 0.5 when curated-positive and matched background pairs are present in equal numbers. Error bars in Panel C represent ±1 SD across final biological contexts; Panels (**A**,**B**) show mean values for each context.

Across PBMC, adult tissue, and adult cell-type contexts, all five metrics collectively showed weak but reproducible statistical visibility of curated TF–target edges relative to strictly matched background pairs. Across the eight final PBMC and adult tissue contexts, the final-context mean AUPRC values for absolute Pearson and absolute Spearman were 0.539 and 0.541, respectively. Across the seven final adult cell-type contexts, the corresponding mean AUPRC values were 0.553 and 0.549. The co-detection odds ratio also showed a positive tendency, with a mean AUPRC of 0.557 in PBMC plus adult tissue contexts and 0.562 in adult cell types. MI and co-expression probability were closer to the 0.5 baseline. Their mean AUPRC values were 0.530 and 0.514, respectively, in PBMC plus adult tissue contexts and 0.544 and 0.515 in adult cell types. These context-level patterns and group-level summaries are shown in Figure 2 and Table 1. Across the group-level summaries, mean AUPRC values ranged from 0.514 to 0.562, with the maximum observed value remaining close to the random-ranking baseline.

These results indicate that curated TF–target edges retained reproducible expression-level association signals after strict matching for target-gene expression level and detection rate. Because their ranking advantage over the 0.5 baseline was modest, the results support statistical visibility rather than strong separation between curated-positive and matched background pairs.

### 3.2. High-Scoring TF–Gene Pairs Show Reverse Consistency

**Figure 3:**
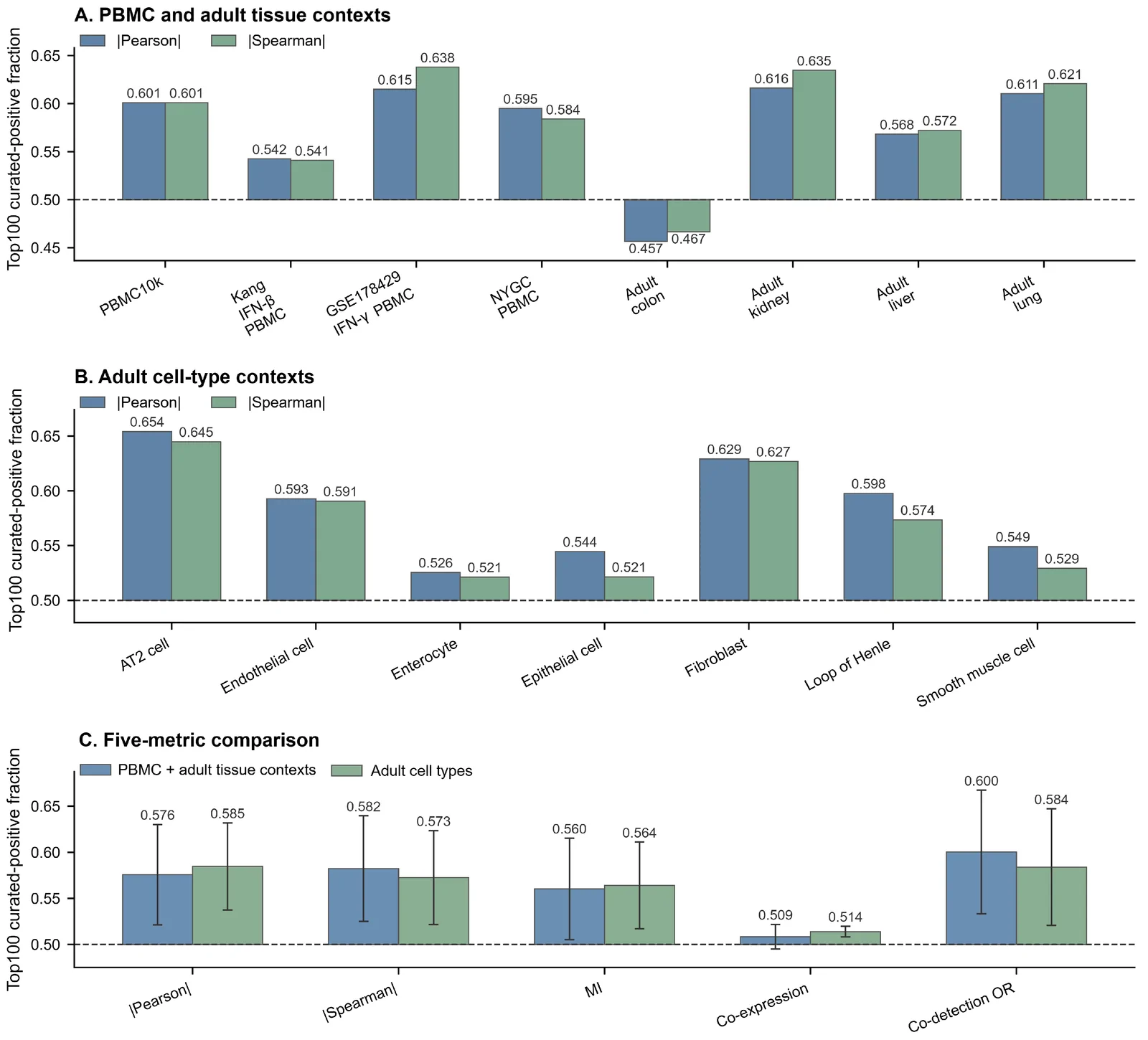
Reverse consistency of high-scoring TF–gene pairs at the fixed Top100 threshold. Panel (**A**) shows the curated-positive fractions for absolute Pearson and absolute Spearman in PBMC and adult tissue contexts; Panel (**B**) shows the same two metrics in adult cell-type contexts. To maintain readability at the context level, the first two panels display only these two correlation metrics. Panel (**C**) reports the equal-weight mean fixed Top100 curated-positive fraction across final biological contexts for all five pair-level metrics in PBMC plus adult tissue contexts and adult cell types. The dashed line indicates the expected balanced baseline of 0.5 when curated-positive and matched background pairs are present in equal numbers. Evaluations with fewer than 100 ranked pairs are missing from fixed Top100 and are excluded from the corresponding means. Error bars in Panel (**C**) represent ±1 SD across final biological contexts; Panels (**A**,**B**) show mean values for each context.

Reverse consistency analysis reused the matched evaluation sets from forward validation across four PBMC datasets, four adult tissues, and seven adult cell types. The control and interferon-stimulated conditions in Kang and GSE178429 were first analyzed separately and then combined to form one final biological context for each dataset before joining the other contexts in cross-context summaries. Because each evaluation set contained equal numbers of curated-positive and matched background pairs, the balanced baseline for the curated-positive fraction was 0.5. The primary analysis used fixed Top100. Evaluations with fewer than 100 ranked pairs were missing at this threshold. Adult colon × intersection contained 96 ranked pairs and therefore had no fixed Top100 estimate, while the other analyzable edge sets for this context were retained in the relevant summaries. In both group-level summaries, the fixed Top100 curated-positive fractions for all five metrics were above 0.5, with larger deviations for absolute Pearson, absolute Spearman, and the co-detection odds ratio. In PBMC plus adult tissue contexts, these values were 0.576, 0.582, and 0.600, respectively; the corresponding values in adult cell types were 0.585, 0.573, and 0.584. MI and co-expression probability also remained slightly above baseline at the group level, with values of 0.560 and 0.509, respectively, in PBMC plus adult tissue contexts and 0.564 and 0.514 in adult cell types. In adult colon, the fixed Top100 fractions for absolute Pearson and absolute Spearman were 0.457 and 0.467, respectively, while their forward AUPRC values were 0.500 and 0.511. Across the valid evaluation sets for this context, fixed Top100 represented 29.8–56.2% of ranked pairs. With proportional top-5% selection, the corresponding fractions were 0.543 and 0.608, indicating that fixed-threshold results were more sensitive to threshold definition in a context with weaker visibility and smaller evaluation sets. The fixed Top100 results and the strict Top500 and top-5% sensitivity analyses are summarized in Figure 3 and Supplementary Table S3.

This analysis supported the forward-validation results from the reverse direction. Within the strictly matched evaluation space, higher-scoring TF–gene pairs were more likely to correspond to curated regulatory relationships. However, the curated-positive fraction does not represent recall of all regulatory edges. High-scoring pairs absent from the databases may reflect co-expression, shared pathway activation, cellular composition, or regulatory relationships not yet captured by curated resources.

### 3.3. High-Scoring Target Genes Show Pathway Coherence

To further assess whether high-scoring curated-positive pairs had an interpretable functional context, Hallmark pathway enrichment was evaluated for their target genes within each biological context. At the PBMC dataset-group level, IL-6/JAK/STAT3 signaling, inflammatory response, allograft rejection, IFN-γ response, hypoxia, complement, and TNFα signaling via NF-κB were repeatedly supported among Top100 target genes. The adult tissue and adult cell-type summaries showed partly different Hallmark patterns, including inflammatory, apoptotic, hypoxic, TNFα/NF-κB, p53, IFN-γ, and mTORC1-related programs, depending on the group.

**Figure 4:**
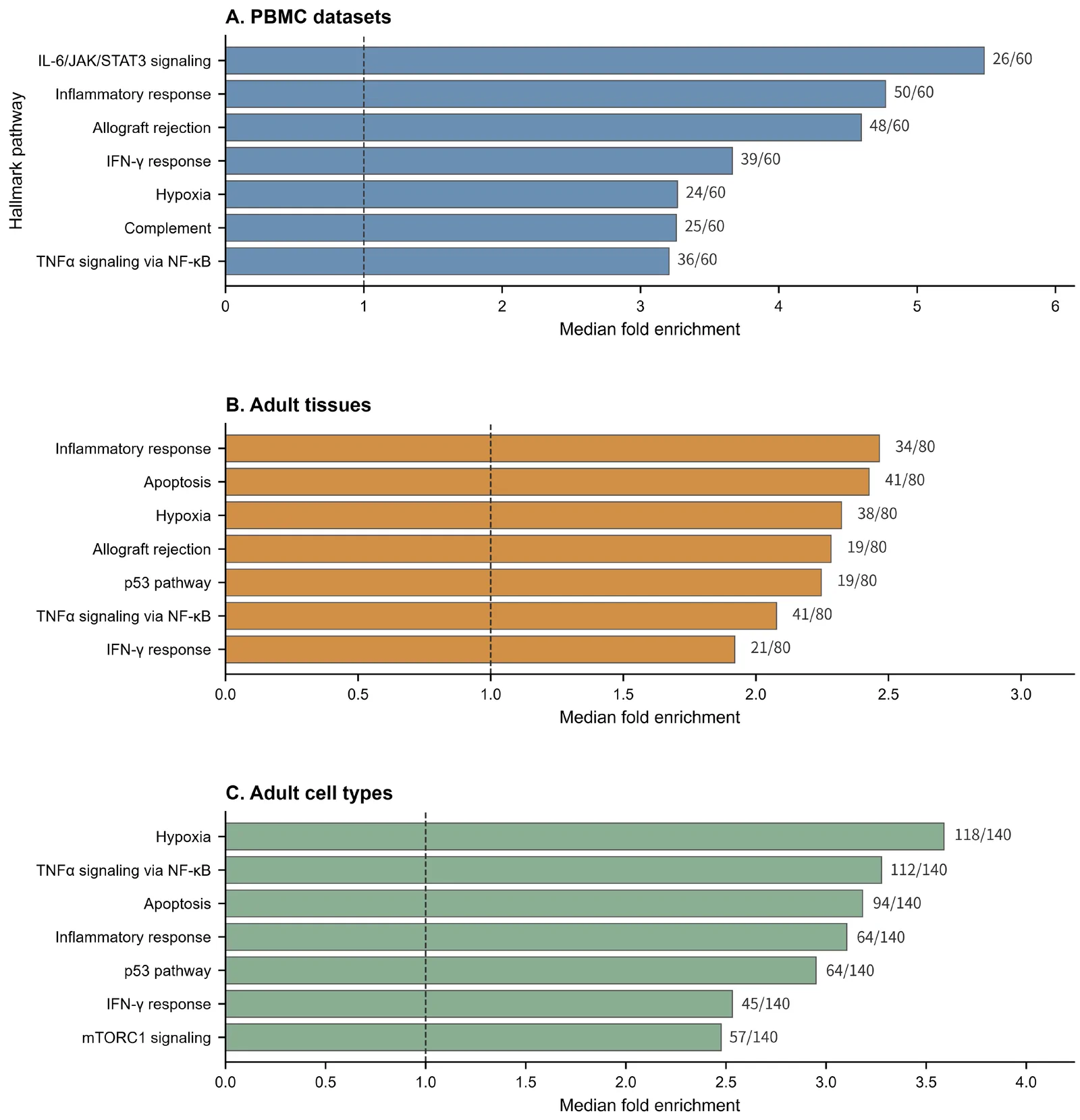
Hallmark pathway enrichment among target genes of high-scoring curated TF–target pairs. Panels (**A**–**C**) show group-level summaries for PBMC datasets, adult tissues, and adult cell types, respectively. Enrichment tests were performed independently within each biological context; the group-level panels summarize analyzable condition × edge-set × metric combinations. The x-axis represents FE. The dashed line marks FE = 1, and values > 1 indicate enrichment of the Hallmark pathway among target genes from high-scoring curated-positive pairs relative to the corresponding background. Each bar represents the median FE across all analyzable combinations. Bar-end labels show supported/analyzable condition × edge-set × metric combinations, where support indicates that the same combination reached FDR < 0.05 in at least 5 of 10 matched-background repeats. For each group, Hallmark pathways from the fixed-Top100 analysis were ranked primarily by support fraction, with median FE used to break ties. The seven highest-ranking pathways were selected for display and are shown in descending order of median FE.

These enrichment patterns indicate context-specific functional coherence among the target genes of high-scoring curated-positive pairs. The corresponding FE values and support counts are provided in Figure 4 and Supplementary Table S4.

## 4. Discussion

This study showed that, after strict matching for target-gene mean expression and detection rate, curated TF–target regulatory edges retained weak but reproducible statistical visibility relative to matched background pairs in single-cell expression matrices. All five pair-level metrics showed visibility above the balanced baseline in the group-level summaries. Signals were more stable for absolute Pearson, absolute Spearman, and the co-detection odds ratio, indicating that statistical metrics differ in their sensitivity to expression-level association patterns in single-cell expression space.

The strength of statistical visibility is influenced by several factors. Post-transcriptional regulation can cause TF protein abundance to diverge from mRNA abundance [3]. Post-translational modifications may also alter TF activity [4]. Chromatin state, pathway activation, temporal delay, cell-state composition, and sparsity in single-cell expression matrices may all modify TF–target expression associations [5]. Because this study evaluated pairwise associations in mRNA expression matrices, some curated edges would not be expected to show strong statistical signals in the current single-cell expression space. This delineates the scope of applicability for expression-level analysis.

Reverse consistency analysis further showed that the curated-positive fraction among fixed Top100 pairs was generally above the balanced baseline of 0.5 at the group level. Adult colon illustrates the context dependence of this tendency. Its weaker forward separation and smaller evaluation sets resulted in broader fixed Top100 coverage, with fixed Top100 fractions below baseline for absolute Pearson and absolute Spearman; both values were above 0.5 under proportional top-5% selection. Hallmark pathway enrichment showed context-specific functional coherence among target genes from high-scoring curated-positive pairs, providing biological context for the statistical signals.

Several limitations should be noted. The analysis depends on existing database annotations, and background pairs were interpreted as matched comparison pairs rather than validated nonregulatory edges. Curated database annotations and LLM-assisted regulatory suggestions differ in their evidence structure. Curated resources provide predefined TF–target edges supported by literature curation or confidence-ranked evidence integration. LLM-assisted outputs may reflect broader textual associations, implicit biological context, and model-specific inference patterns. The present analysis therefore evaluates the expression-level visibility of curated prior knowledge rather than the validity of any specific automated prediction system. All five metrics measure expression-level statistical association and cannot distinguish direct regulation, indirect co-expression, or associations driven by cell state. The set of analyzable positive edges also varies across datasets, conditions, and edge sets. Kang IFN-β PBMC and GSE178429 IFN-γ PBMC were evaluated only with the TRRUST–DoRothEA A/B consensus intersection. These datasets therefore assessed the statistical visibility of the same consensus network across control and interferon-stimulated conditions and across different PBMC datasets; they did not independently test robustness across different edge-set definitions. The latter was assessed through the four-edge-set analyses in PBMC10k, NYGC multimodal PBMC, and the HCL contexts. The conclusions should therefore be limited to statistical visibility and reverse consistency rather than causal regulatory validation.

## 5. Conclusions

Across four PBMC datasets, four adult tissues, and seven adult cell types, curated TF–target regulatory edges showed weak but reproducible statistical visibility relative to background gene pairs strictly matched for target-gene expression level and detection rate. The five pair-level association metrics captured this visibility from different perspectives, with absolute Pearson, absolute Spearman, and the co-detection odds ratio showing the most stable signals. These findings indicate that biologically supported regulatory relationships do not always form strong and stable separation signals in single-cell expression space, but they can retain measurable expression-level statistical traces.

Overall, single-cell expression associations can provide statistical clues and functional-context evidence for TF–target regulatory relationships, but their signal strength and interpretability have inherent limits. These results support the limited observability of curated regulatory edges in single-cell expression space and the reverse consistency between high-scoring pairs and curated regulatory knowledge. For applications of large language models and automated analytical tools to regulatory relationship interpretation, the findings indicate that outputs should not be understood solely in terms of model performance but should also be evaluated in light of the limited ability of single-cell expression data to represent genuine regulatory relationships. This study provides an empirical reference for interpreting TF–target expression associations in specific cell states or stimulation conditions and for evaluating the performance boundaries of automated tools.

## Figures and Tables

**Table 1:** Forward-validation performance across final biological contexts. The “Contexts” column indicates the number of final biological contexts in each group. AUPRC, AUROC, and Cliff’s delta are reported as equal-weight means across contexts. “AUPRC > 0.5” indicates the number of contexts with a mean AUPRC above the balanced baseline. Under the balanced 1:1 design, the expected AUPRC for random ranking is 0.5.

Context Group	Metric	Contexts	AUPRC	AUROC	AUPRC > 0.5	Cliff’s Delta
PBMC + adult tissues	Absolute Pearson	8	0.539	0.533	7/8	0.067
PBMC + adult tissues	Absolute Spearman	8	0.541	0.534	8/8	0.069
PBMC + adult tissues	Mutual information	8	0.530	0.524	8/8	0.048
PBMC + adult tissues	Co-expression probability	8	0.514	0.514	8/8	0.028
PBMC + adult tissues	Co-detection odds ratio	8	0.557	0.550	8/8	0.101
Adult cell types	Absolute Pearson	7	0.553	0.546	7/7	0.093
Adult cell types	Absolute Spearman	7	0.549	0.544	7/7	0.088
Adult cell types	Mutual information	7	0.544	0.539	7/7	0.079
Adult cell types	Co-expression probability	7	0.515	0.515	7/7	0.031
Adult cell types	Co-detection odds ratio	7	0.562	0.564	7/7	0.128

## Data Availability

All single-cell datasets analyzed in this study were obtained from publicly available resources, including the 10x Genomics public data portal, GEO accession GSE178429, and the published PBMC and HCL/ZJU adult tissue and adult cell-type data sources cited in the main text. Curated TF–target relationships were obtained from TRRUST v2 and DoRothEA A/B. Pathway annotations used for pathway coherence analysis were obtained from the MSigDB Hallmark collection. Analysis scripts and processed summary tables are available at https://github.com/wenqing367/TF-target-statistical-visibility (accessed on 17 August 2026).

## Supplementary Materials

The following supporting information can be downloaded at: https://www.mdpi.com/article/10.3390/genes17091110/s1, Supplementary Table S1, dataset, context, condition, and edge-set coverage; Supplementary Table S2, strict matched-background quality-control summary; Supplementary Table S3, reverse-consistency summaries for the fixed Top100 analysis and the strict Top500 and top-5% sensitivity analyses; Supplementary Table S4, Hallmark pathway values displayed in Figure 4.

## Abbreviations

The following abbreviations are used in this manuscript:

AUPRC	area under the precision–recall curve
AUROC	area under the receiver operating characteristic curve
FDR	false discovery rate
FE	fold enrichment
GEO	Gene Expression Omnibus
HCL	Human Cell Landscape
IFN-β	interferon beta
IFN-γ	interferon gamma
IL-6	interleukin-6
JAK	Janus kinase
MI	mutual information
mTORC1	mechanistic target of rapamycin complex 1
MSigDB	Molecular Signatures Database
NF-κB	nuclear factor kappa-light-chain-enhancer of activated B cells
NYGC	New York Genome Center
PBMC	peripheral blood mononuclear cell
scRNA-seq	single-cell RNA sequencing
SD	standard deviation
STAT3	signal transducer and activator of transcription 3
TF	transcription factor
TNFα	tumor necrosis factor alpha
