# Supplementary material for: Statistical Visibility of Curated TF–Target Regulatory Relationships and Reverse Consistency of Top-Ranked TF–Gene Pairs in Single-Cell Expression Data
Source: Genes (Basel). 2026 Sep 12;17(9):1110. doi: 10.3390/genes17091110 (PMC13606148; doi:10.3390/genes17091110)
Supplement: Supplementary file 1 [file genes-17-01110-s001.zip › genes-4538211-supplementary.pdf]

Supplementary Materials

Supplementary Materials for: Statistical visibility of curated TF–target regulatory relationships and reverse consistency of top-ranked TF–gene pairs in single-cell expression data

These supplementary tables report dataset coverage, strict matched-background quality control, reverse-consistency summaries and sensitivity analyses, and the Hallmark pathway values are displayed in Figure 4.

Abbreviations: AUPRC, area under the precision–recall curve; AUROC, area under the receiver operating characteristic curve; SMD, standardized mean difference; FDR, false discovery rate; MI, mutual information; FE, fold enrichment; NA, not analyzable at the specified threshold.

S1. Dataset, context, condition, and edge-set coverage

Table S1 summarizes the 15 final biological contexts and 56 condition × edge-set analysis units. Final biological contexts were used for main-figure and cross-context summaries; edge sets and matched-background repeats were not treated as biological replicates.

Table S1. Dataset, context, condition, and edge-set coverage.

Biological group	Dataset/final context	Condition(s)	Available edge sets	Analysis units	Retained positives	Matching coverage	Repeats
PBMC datasets	PBMC10k	unstratified	All four	4	1079-6855	97.1%-98.4%	10
PBMC datasets	Kang IFN-β PBMC	ctrl; stim	intersection	2	294-301	89.9%-94.1%	10
PBMC datasets	GSE178429 IFN-γ PBMC	ctrl 6 h; IFN-γ 6 h	intersection	2	405-805	89.3%-90.8%	10
PBMC datasets	NYGC multimodal PBMC/NYGC PBMC	unstratified	All four	4	1041-6568	96.7%-98.6%	10
Adult tissues	HCL/ZJU adult tissue/Adult colon	unstratified	All four	4	48-168	96.9%-98.0%	10

Biological group	Dataset/final context	Condition(s)	Available edge sets	Analysis units	Retained positives	Matching coverage	Repeats
Adult tissues	HCL/ZJU adult tissue/Adult kidney	unstratified	All four	4	213-997	93.3%-94.2%	10
Adult tissues	HCL/ZJU adult tissue/Adult liver	unstratified	All four	4	76-304	87.1%-90.4%	10
Adult tissues	HCL/ZJU adult tissue/Adult lung	unstratified	All four	4	229-983	93.1%-95.5%	10
Adult cell types	HCL/ZJU adult cell type/AT2 cell	unstratified	All four	4	157-718	95.4%-98.0%	10
Adult cell types	HCL/ZJU adult cell type /Endothelial cell	unstratified	All four	4	242-1073	90.6%-93.7%	10
Adult cell types	HCL/ZJU adult cell type /Enterocyte	unstratified	All four	4	57-265	91.7%-92.8%	10
Adult cell types	HCL/ZJU adult cell type /Epithelial cell	unstratified	All four	4	140-646	92.1%-93.3%	10
Adult cell types	HCL/ZJU adult cell type /Fibroblast	unstratified	All four	4	186-713	94.0%-95.9%	10
Adult cell types	HCL/ZJU adult cell type/Loop of Henle	unstratified	All four	4	141-752	91.2%-92.8%	10
Adult cell types	HCL/ZJU adult cell type /Smooth muscle cell	unstratified	All four	4	192-793	88.5%-90.6%	10

The label “All four” denotes TRRUST, DoRothEA A/B, their intersection, and their union. The label “unstratified” indicates a single analysis condition in which all available cells from the corresponding dataset or context were analyzed together, rather than being separated by stimulation or treatment condition. The intersection comprises TF–target edges shared by TRRUST and DoRothEA A/B. Kang IFNβ PBMC and GSE178429 IFNγ PBMC were evaluated only with this conservative consensus intersection; their control and interferon-stimulated conditions were assessed separately using the same edge set. This design assessed the statistical visibility of the same consensus network across conditions and across the two PBMC datasets. PBMC10k, NYGC multimodal PBMC, and the HCL adult tissue and cell-type contexts were evaluated with all four predefined edge sets to assess robustness to the

definition of the prior network. Across all contexts, the design comprised 56 condition × edge-set analysis units, each evaluated using 10 matched-background repeats.

S2. Strict matched-background quality-control summary

Table S2 summarizes the strict matched-background checks across all 56 analysis units. All units completed 10 repeats with equal positive and background counts, no final failed or relaxed matches, and no duplicate, overlap, or curated-union leakage in the retained background pairs.

Table S2. Strict matched-background quality-control summary.

QC item	Value
Condition × edge-set analysis units	56
Matched-background repeats per unit	10
Retained positive edges per unit	48-6855
Matching coverage across units	87.1%-98.6%
Equal positive/background counts in every repeat	56/56
Duplicated background pairs	0
Positive-background overlap	0
Background leakage into curated-edge union	0
Maximum absolute SMD, target mean expression	0.065
Maximum absolute SMD, target detection rate	0.012

SMD denotes standardized mean difference. Leakage denotes a matched background pair that also appears in the union of curated edges. Matching coverage is the proportion of curated-positive edges retained after strict matched-background construction.

S3. Reverse-consistency main and sensitivity summary

The primary reverse-consistency analysis used fixed Top100. Table S3 reports the equal-weight summary across final biological contexts corresponding to Figure 3C, together with the strict Top500 and top-5% sensitivity analyses.

Table S3. Reverse-consistency summaries for the fixed Top100 analysis and the strict Top500 and top-5% sensitivity analyses.

Analysis threshold	Context group	Metric	Valid final contexts	Mean curated-positive fraction	Contexts > 0.5	Delta vs 0.5
Fixed Top100	PBMC + adult tissues	|Pearson|	8	0.576	7/8	0.076
Fixed Top100	PBMC + adult tissues	|Spearman|	8	0.582	7/8	0.082
Fixed Top100	PBMC + adult tissues	MI	8	0.560	6/8	0.060
Fixed Top100	PBMC + adult tissues	Coexpression probability	8	0.509	6/8	0.009
Fixed Top100	PBMC + adult tissues	Codetection odds ratio	8	0.600	7/8	0.100
Fixed Top100	Adult cell types	|Pearson|	7	0.585	7/7	0.085
Fixed Top100	Adult cell types	|Spearman|	7	0.573	7/7	0.072

Analysis threshold	Context group	Metric	Valid final contexts	Mean curated-positive fraction	Contexts > 0.5	Delta vs 0.5
Fixed Top100	Adult cell types	MI	7	0.564	7/7	0.064
Fixed Top100	Adult cell types	Coexpression probability	7	0.514	7/7	0.014
Fixed Top100	Adult cell types	Codetection odds ratio	7	0.584	6/7	0.084
Strict Top500	Adult cell types	Codetection odds ratio	7	0.541	7/7	0.041
Strict Top500	Adult cell types	Coexpression probability	7	0.508	7/7	0.008
Strict Top500	Adult cell types	MI	7	0.528	6/7	0.028
Strict Top500	Adult cell types	|Pearson|	7	0.530	7/7	0.030
Strict Top500	Adult cell types	|Spearman|	7	0.530	6/7	0.030
Strict Top500	PBMC + adult tissues	Codetection odds ratio	7	0.541	7/7	0.041
Strict Top500	PBMC + adult tissues	Coexpression probability	7	0.510	6/7	0.010
Strict Top500	PBMC + adult tissues	MI	7	0.522	7/7	0.022
Strict Top500	PBMC + adult tissues	|Pearson|	7	0.528	7/7	0.028
Strict Top500	PBMC + adult tissues	|Spearman|	7	0.527	7/7	0.027
top-5%	Adult cell types	Codetection odds ratio	7	0.626	6/7	0.126
top-5%	Adult cell types	Coexpression probability	7	0.526	5/7	0.026
top-5%	Adult cell types	MI	7	0.616	6/7	0.116
top-5%	Adult cell types	|Pearson|	7	0.643	6/7	0.143
top-5%	Adult cell types	|Spearman|	7	0.618	7/7	0.118

Analysis threshold	Context group	Metric	Valid final contexts	Mean curated-positive fraction	Contexts > 0.5	Delta vs 0.5
top-5%	PBMC + adult tissues	Codetection odds ratio	8	0.632	8/8	0.132
top-5%	PBMC + adult tissues	Coexpression probability	8	0.516	7/8	0.016
top-5%	PBMC + adult tissues	MI	8	0.578	7/8	0.078
top-5%	PBMC + adult tissues	|Pearson|	8	0.591	8/8	0.091
top-5%	PBMC + adult tissues	|Spearman|	8	0.609	8/8	0.109

Adult colon × intersection had evaluation N = 96; fixed Top100 was therefore NA and was excluded from fixed Top100 averages. The other analyzable adult-colon edge sets were retained. The expected curated-positive fraction under the balanced design is 0.5.

Strict Top500 required evaluation N ≥ 500; smaller units were NA. Unit-level estimates were aggregated within final contexts, which were then averaged with equal weight. Strict Top500 was analyzable in 19/28 condition × edge-set units in each group (38/56 overall) and 14/15 contexts. Top-5% retained the actual top-N and was analyzable in all 56 units; median final-context top N was 61.0 for PBMC plus adult tissues and 45.5 for adult cell types. Fixed Top100 occupied a large fraction of several small sets: Enterocyte × intersection (87.7%; N = 114), adult liver × intersection (65.8%; N = 152), and adult colon × DoRothEA A/B (56.2%; N = 178). Adult colon had no valid strict Top500 edge set; its top-5% final-context means were 0.543 (absolute Pearson) and 0.608 (absolute Spearman). Analysis units, edge sets, and matched-background repeats were not treated as biological replicates.

S4. Hallmark pathway-enrichment numerical summary

Table S4 reports only the Hallmark pathways displayed in Figure 4, summarized separately for PBMC datasets, adult tissues, and adult cell types.

Table S4. Hallmark pathway values displayed in Figure 4.

Group	Pathway	Median FE	Supported combinations	Analyzable combinations	Support fraction
PBMC datasets	IL-6/JAK/STAT3 signaling	5.485	26	60	0.433
PBMC datasets	Inflammatory response	4.772	50	60	0.833
PBMC datasets	Allograft rejection	4.596	48	60	0.800
PBMC datasets	IFN-γ response	3.664	39	60	0.650
PBMC datasets	Hypoxia	3.267	24	60	0.400
PBMC datasets	Complement	3.260	25	60	0.417
PBMC datasets	TNFα signaling via NF-κB	3.206	36	60	0.600
Adult tissues	Inflammatory response	2.466	34	80	0.425
Adult tissues	Apoptosis	2.427	41	80	0.512
Adult tissues	Hypoxia	2.323	38	80	0.475
Adult tissues	Allograft rejection	2.283	19	80	0.237
Adult tissues	p53 pathway	2.246	19	80	0.237
Adult tissues	TNFα signaling via NF-κB	2.078	41	80	0.512
Adult tissues	IFN-γ response	1.920	21	80	0.263
Adult cell types	Hypoxia	3.589	118	140	0.843
Adult cell types	TNFα signaling via NF-κB	3.277	112	140	0.800
Adult cell types	Apoptosis	3.182	94	140	0.671
Adult cell types	Inflammatory response	3.103	64	140	0.457
Adult cell types	p53 pathway	2.950	64	140	0.457
Adult cell types	IFN-γ response	2.531	45	140	0.321
Adult cell types	mTORC1 signaling	2.476	57	140	0.407

Only Hallmark gene sets are included. Support is counted for each condition × edge-set × metric combination and requires FDR < 0.05 in at least 5 of 10 matched-background repeats for that combination. Median FE is calculated across all analyzable combinations without significance filtering. Group-level summaries do not imply that every context within a group shares the same pathway.
